# Nightmares and Sleep Disturbances in Children with PTSD: A Polysomnographic and Actigraphy Approach Evaluation

**DOI:** 10.3390/jcm12206570

**Published:** 2023-10-17

**Authors:** Julie Rolling, Juliette Rabot, Eve Reynaud, Oriane Kolb, Patrice Bourgin, Carmen M. Schroder

**Affiliations:** 1 Department of Child and Adolescent Psychiatry, Strasbourg University Hospital, 67091 Strasbourg, France; juliette.rabot.med@ssss.gouv.ca (J.R.); oriane.kolb@chru-strasbourg.fr (O.K.); schroderc@unistra.fr (C.M.S.); 2 Regional Center for Psychotraumatism Great East, Strasbourg University Hospital, 67091 Strasbourg, France; 3 Sleep Disorders Center, International Research Center for ChronoSomnology, Strasbourg University Hospital, 67091 Strasbourg, France; eve.reynaud@chru-strasbourg.fr (E.R.); pbourgin@unistra.fr (P.B.); 4 CNRS UPR 3212, Institute for Cellular and Integrative Neurosciences, University of Strasbourg, 67081 Strasbourg, France; 5 Center for Research, Integrated University Health and Social Services Center (CIUSSS) Nord-de-l’Île-de-Montréal, Montréal, QC H2M 2W1, Canada; 6 Department of Psychiatry & Addictology, University of Montreal, Montreal, QC H3T 1C5, Canada; 7 CNRS, INSERM, Centre de Recherche en Neurosciences de Lyon CRNL U1028 UMR5292, Forgetting, Université Claude Bernard Lyon 1, 69500 Bron, France

**Keywords:** post-traumatic stress disorder, sleep disturbances, nightmares, video-polysomnography, children

## Abstract

Rationale: Sleep disturbances (insomnia and nightmare symptoms) are the most sensitive and persistent symptoms of pediatric post-traumatic stress disorder (PTSD). Untreated, these sleep disturbances (SD) associated with PTSD are predictive of PTSD persistence and increased psychiatric complications. The aim of this study was to evaluate sleep and circadian rhythms in children with PTSD under both laboratory and ecological conditions in comparison with a control population and to test for the first time the hypothesis that SD and circadian rhythms are positively correlated with PTSD severity and its comorbidities. Method: This prospective pilot study evaluated PTSD, SD (insomnia, nightmares), and sleep-wake rhythms in 11 children with PTSD (aged 3–18), compared with the age and sex-matched control groups. Assessment of PTSD and subjective and objective measures of sleep and sleep-wake rhythms (questionnaires, 24-h in-laboratory video-polysomnography, 15-day at-home actigraphy recording) were performed between 1 and 6 months after the traumatic event. Results: Children with PTSD had higher sleep fragmentation (increased wake-after-sleep onset, increased number of sleep stage changes) compared to controls, with a change in sleep microarchitecture (micro-arousal index at 14.8 versus 8.2, *p* = 0.039). Sleep fragmentation parameters correlated with PTSD symptomatology, insomnia, and post-traumatic nightmare severity. The within-group comparison revealed a better sleep architecture in the controlled (sleep laboratory) than in the ecological condition (at home) (total sleep time 586 versus 464 min, *p* = 0.018). Conclusions: Sleep and rhythm disturbances are strongly associated with PTSD in children. The assessment of SD in children with PTSD should be carried out systematically and preferentially under ecological conditions, and management of SD should integrate the environment (environmental design, psycho-education for the children and their parents) more fully into therapy focused on sleep and trauma.

## 1. Introduction

### 1.1. Post-Traumatic Stress Disorder in Children and Adolescents

After a traumatic event, around 1.3 to 12.2% of exposed adults develop symptoms of post-traumatic stress disorder (PTSD) over a lifetime, and 0.2 to 3.8% develop these symptoms over one year [1]. Studies in child psychiatry are rarer but consistent with results obtained in adults. In the general population, they show a prevalence of exposure to traumatic events in childhood and adolescence of 31.1% and a prevalence of PTSD of 7.8% at the age of 18 (studies carried out on 2232 subjects) [2], with an overall average rate of PTSD of 15.9%, depending on the type of trauma and sex [3], and of 3.4% for the PTSD-complex recently defined by the ICD-11 [4]. According to a recent study of 866 children exposed to the Nice attacks, up to 71% of children in clinical populations followed by child psychiatrists suffer from PTSD [5]. Once present, post-traumatic symptomatology largely affects the child’s cognitive and psycho-affective development, with a global and significant impact on mental and physical health in the medium and long term.

### 1.2. Sleep Disturbances in Children and Adolescents with PTSD

Sleep disturbances are the earliest, most sensitive, and persistent symptoms of PTSD in children. They may appear immediately after the event, even before the development of PTSD [6], or fall within the scope of PTSD according to the DSM-5 definition [7]: daytime and nocturnal intrusion symptoms (distressing dreams in which the content and/or affect of the dream are related to the traumatic event(s), i.e., trauma-related nightmares), psychic and/or behavioral avoidance, negative alterations in cognitions and mood, neurovegetative hyperactivation with sleep disturbances (insomnia symptomatology) [8].

Among sleep disturbances, the most specific symptoms are the onset or increase in insomnia symptoms and parasomnias, particularly nightmares. A higher rate of other types of parasomnias associated with nightmares, mainly night terrors, is also observed in children with PTSD [9]. Sleep disturbances as a whole affect more than 50% of children and adolescents one year after an initial trauma [6]. Among these disturbances, posttraumatic nightmares have specific characteristics [10] and are pathognomonic symptoms of PTSD with a prevalence rate after a traumatic event that varies from 50% to 80% [11,12], rising to 100% after a major trauma [13].

In children and adolescents, sleep disturbances may not only predict the development, severity [14], and persistence of PTSD [11], but they also tend to become chronic and are associated with the development of PTSD comorbidities such as depression and anxiety [15,16]. More generally, sleep disturbances can also increase general distress, substance use, and suicidal behavior [6]. Finally, it has also been shown that sleep disturbances in adolescents are associated with a decreased response rate to the gold standard therapy for PTSD: trauma-focused cognitive behavioral therapy (Tf-CBT) [17].

These sleep disturbances (insomnia symptoms and parasomnias) lead to changes in sleep patterns (increased sleep onset latency, more fragmented sleep of poorer overall quality) [6]. In this respect, studies of polysomnographic recordings in the adult population highlight an increase in sleep onset latency, a decrease in sleep efficiency, and a higher fragmentation of sleep with an increase in wake-after-sleep onset (WASO), as well as arousal and micro-arousal indexes [18,19,20]. For REM sleep, the average duration of continuous REM sleep was found to be decreased, but the number of REM sleep periods increased [21] with a concomitant increase in eye movement density [18,22]. The few objective studies conducted in children, some of which use actigraphy recordings, confirm studies using subjective assessments [6], demonstrating a high prevalence of symptoms on the insomnia spectrum in pediatric PTSD: sleep initiation disturbances, early morning awakenings, fragmented sleep with multiple nocturnal awakenings, mainly during REM sleep [6,23,24].

The majority of studies using objective assessment through polysomnographic recordings have been realized in adults with PTSD, usually veterans, i.e., a very different population from a child psychiatric population with PTSD [18,19,20,21]. Furthermore, the rare studies evaluating the sleep of children with PTSD are mainly based on subjective evaluations of the child’s sleep [6] or objective measurements from actigraphy [6,23,24]. In addition, there is no conclusive work to date on possible associated sleep-wake rhythm disturbances (i.e., circadian rhythm disturbances) in children with PTSD, while research published on adults indicates a vulnerability of evening people to more severe stress reactions [25]. However, the study of sleep and circadian rhythm disturbances in PTSD is essential because there are common pathophysiological bases between sleep disturbances and posttraumatic symptoms, and effective treatments targeting sleep disturbances in PTSD can improve sleep and overall PTSD symptomatology and alleviate PTSD symptom trajectory.

These common pathophysiological bases are strongly linked to the activation of the autonomic nervous system (ANS), which is closely linked to the regulation of the various stages of sleep and the onset of PTSD symptoms [9,20]. Among others, the Locus Coeruleus (LC) is involved in the regulation of the alternation of the sleep-wake cycle and REM sleep onset [26]. The LC is one of the main noradrenergic structures with projections to the amygdala, the hippocampus, the prefrontal cortex, the raphe nuclei, and the paraventricular nuclei of the hypothalamus. One hypothesis would be that REM sleep fragmentation would be linked to a disinhibition of noradrenergic activity. Recent work has similarly highlighted an activation of the amygdala during REM sleep, which would strengthen the physiological hypothesis of a possible role in the bidirectional interaction between the amygdala, REM sleep, and PTSD-related sleep disturbances [27]. Finally, prazosin, an alpha-adrenergic antagonist, has shown some effect on childhood PTSD-associated nightmares, although the level of evidence remains insufficient to date [28,29]. In addition, psychotherapies specifically targeting post-traumatic nightmares, such as mental imagery rehearsal therapy, have also shown their effectiveness on both sleep disturbances and PTSD symptomatology [30].

Overall, the scientific literature on children with PTSD suggests (1) that sleep disturbances, particularly nightmares, are frequent, early onset but persisting and disabling symptoms of PTSD; (2) that SD in children with PTSD have a very significant impact on the mental health of children, and that they are predictive of the persistence of PTSD in the long term, as well as an increase in comorbid disorders; (3) that sleep disturbances are associated with a reduced response to standard therapy for PTSD and (4) that psychotherapeutic and pharmacological treatment of sleep disturbances may improve both sleep disturbances in PTSD as well as certain symptoms of PTSD, though data on the latter are preliminary to date.

Based on these findings and hypotheses, the main aim of our study was (i) to objectively evaluate sleep and circadian rhythms in children with PTSD compared to a control population, with the hypothesis that children with PTSD present more sleep disturbances (increased sleep onset latency, decreased total sleep time, increase in the number of nocturnal awakenings and increase in awake time within the sleep period) than controls, whether in laboratory or ecological conditions and (ii) to evaluate the correlations between the objective sleep and circadian rhythm parameters of children with PTSD and clinical and subjective parameters of PTSD, with the hypothesis that sleep and circadian rhythm disturbances are positively correlated with PTSD severity and PTSD comorbidities.

## 2. Materials and Methods

### 2.1. Participants and Study Setting

Inclusions of children took place from 4 January 2021 to 3 May 2021 at the Sleep Disorders Center/International Center for Research on ChronoSomnology (CIRCSom) at Strasbourg University Hospitals. To carry out this research, children between the ages of 3 and 18 were recruited from consultations within the Department of Child and Adolescent Psychiatry or via the Regional Center for Psychotraumatology. All children had an established diagnosis of PTSD according to DSM-5 criteria [7] and significant sleep disturbances within the insomnia spectrum (criteria according to the International Classification of Sleep Disorders, Third Edition (ICSD-3, 2014, [9]). Pediatric PTSD arose either following a single traumatic exposure (type 1 trauma as defined) or repeated traumatic exposures (type 2 trauma as abuse, war) [31]. The last traumatic exposure had to have occurred between 6 months and one year prior to recruitment. Children with PTSD on medication were excluded. A total of 11 participants with PTSD between the ages of 3 and 18 years, with no intellectual disabilities and no significant sleep disturbances prior to the traumatic event, were included in this protocol. Controls were used to compare objective sleep parameters derived from PSG. They were recruited from patients hospitalized in the sleep unit for sleep disorders (severe insomnia, parasomnia, restless leg syndrome) requiring PSG exploration, excluding children with severe obstructive sleep apnea syndrome. Controls were matched for age and sex.

The study has been validated by the local ethics committee (acceptance number: CE-2023-76).

This pilot, prospective, and comparative study assessed sleep and sleep-wake rhythms, both in the laboratory and under ecological conditions in the children’s homes. The sleep laboratory visit covered 25 h of hospitalization at CIRCSom. It included a subjective assessment of psychotrauma, sleep, and circadian rhythms using validated auto- and hetero-questionnaires. Objective sleep and rhythm parameters were obtained by video polysomnographic recording (PSG) during the night of hospitalization. Consecutively, rest-activity rhythms were assessed under ecological conditions at the children’s homes using a wrist-worn actigraph (Motionwatch^®^, Cambridge Neurotechnology, Cambridge, UK) for 14 days following hospitalization in order to compare sleep in the laboratory versus ecological conditions. A sleep diary was filled out over the entire period of actigraphy recording.

### 2.2. Variables and Assessment Measures

During hospitalization, each participant completed a set of self-questionnaires for the child and the accompanying parent. The aim of this double evaluation was to explore factors that could increase the child’s PTSD or sleep symptomatology, such as the existence of a double traumatic exposure between child and parent(s) or the existence of sleep disturbances in the parent(s).

*Assessment of PTSD and its comorbidities*

PTSD symptoms in children were assessed using the Child Post-Traumatic Stress Reaction Index (CPTS-RI) diagnostic scale [32] and the Child PTSD Checklist (CPC). The CPTS-RI is a self-administered questionnaire made up of DSM-derived PTSD criteria with 20 items to confirm the diagnosis of PTSD, as well as its level of severity. A total score of 12–24 is associated with mild PTSD, 25–39 with moderate, 40–59 with severe, and over 60 with very severe [32]. The CPC is a 40-item questionnaire developed for children aged 7 to 18 [33]. A score of 20 (or more) on items 14–34 indicates a probable diagnosis of PTSD. A score of 4 (or more) on items 35–40 indicates functional impairment and a probable need for treatment.

Screening for parental PTSD is performed using the Trauma Screening Questionnaire (TSQ), a brief 10-item self-report measure [34]. This screening tool is validated in French and is commonly used in Regional Psychotrauma Centers in France. Each item is derived from DSM-IV criteria. The TSQ has a threshold strictly above 5.

Childhood depression, a frequent comorbidity of PTSD, was assessed using the Child Depression Inventory (CDI) [35]. The CDI is a validated scale for assessing and monitoring depression in children aged 7 to 17. The score ranges from 0 to 54: the higher the score, the more severe the level of depression.

Parental anxiety was assessed using the State-Trait Anxiety Inventory (STAI), a self-report anxiety scale [36]. The anxiety-treatment (AT) part of the STAI was used. It reflects the usual emotional state (STAI form Y-B). The total score indicates the severity of the general state of anxiety, ranging from 20 to 80. A score above 35 indicates the presence of anxiety.

*Subjective assessment of sleep and circadian rhythms*

Each participant’s sleep and circadian rhythms were assessed using subjective (self-questionnaires) and objective (measurements) parameters during hospitalization (laboratory-controlled condition) and at home (ecological condition).

The Insomnia Severity Index (ISI) is a 5-question self-questionnaire assessing the severity of sleep difficulties 1 month prior to assessment. The total score is used to specify the degree of insomnia: no insomnia (0–7), sub-clinical insomnia symptoms (8–14), moderate insomnia (15–21), and severe insomnia (22–28) [37]. The Sleep Disturbance Scale for Children (SDSC) (3 versions exist depending on the child’s age: 6 months to 4 years, 4 to 16 years, and over 16 years) screens for various sleep disturbances using 24 questions divided into 5 categories [38]. A total score of over 45 (items 1 to 25) out of 125 indicates significant sleep disturbances. Sub-scores corresponding to the 5 main sleep disturbances exist, with validated cut-offs: a score higher than 21 out of 35 indicates significant insomnia symptomatology; a score higher than 17 out of 35 indicates significant parasomnia; a score higher than 12 out of 25 indicates significant respiratory problems; a score higher than 11 out of 15 indicates non-restorative sleep and a score higher than 5 out of 15 indicates excessive daytime sleepiness.

The Nightmare Distress Questionnaire (NDQ) is a 13-item self-administered questionnaire measuring nightmare distress [39]. The total score can range from 0 to 52, with an overall threshold above 20 indicating significant distress. Three sub-components exist: the ‘fear’ sub-component with a critical threshold of 10, the ‘interference’ sub-component with a critical threshold of 7, and the ‘premonition’ sub-component with a critical threshold of 4. Semi-standardized questions designed by our team with items specifically targeting nightmares and post-traumatic parasomnias have been added.

The Children’s Chronotype Questionnaire (CCTQ) is a child-friendly version (under 12 years) [40] of the Munich ChronoType Questionnaire (MCTQ) for assessing chronotype. The MCTQ [41] is a self-report questionnaire that distinguishes between work/school days and free days. Measures of circadian rhythm include sleep schedules (bedtime, sleep onset time, sleep latency, and wake-up time), assessed separately for free days versus school days. These measurements are used to calculate chronotype, sleep debt, and social jet lag.

The Pittsburgh Sleep Quality Index (PSQI) used for parents is a self-administered questionnaire assessing sleep quality based on 7 components scored from 0 to 3 [42]. The overall score can vary from 0 to 21, with 0 indicating no difficulties and 21 indicating major sleep difficulties.

*Objective assessment of sleep and circadian rhythms*
*Actigraphy*

The actimeter is a wristwatch-like device containing a piezoelectric accelerometer that measures the intensity, quantity, and duration of physical movements in all directions (Motionwatch^®^, Cambridge Neurotechnology, UK; software (MotionWare 1.2.47), thus assessing the rest-activity rhythms under ecological conditions. The actigraph is worn day and night over a 15-day period alongside a subjective assessment using a sleep diary. Activity is recorded in 1-min epochs, with the use of an algorithm to extrapolate the sleep-wake rhythm, providing an objective estimate of various sleep and circadian rhythms parameters, including total sleep time (TST), sleep onset latency (SOL), wake after sleep onset (WASO) and sleep efficiency. Non-parametric analyses allow for the evaluation of circadian rhythm measurements that can be interpreted as indicators of possible shifts (phase advances or delays) of sleep-wake rhythms, namely the onset of the 5 h of minimum activity over 24 h (L5 onset), the onset of the 10 most active continuous hours over 24 h (M10 onset), the relative amplitude (RA), intraday variability (IV), and interday stability (IS). An RA close to 0 indicates a lack of differentiation between day/night activity and rest, while an RA close to 1 indicates good day/night circadian amplitude. IV reflects the variability of rhythms recorded during a single day, and IS reflects the reproducibility of the rhythm from one day to the next over time. An IS close to 1 indicates a sleep-wake rhythm highly reproducible from one day to the next, whereas an IS close to 0 indicates an instable sleep-wake rhythm. Actigraphy has been validated as a measure of sleep quality for all ages included in this protocol [43,44].

*Polysomnography*

**Figure 1:**
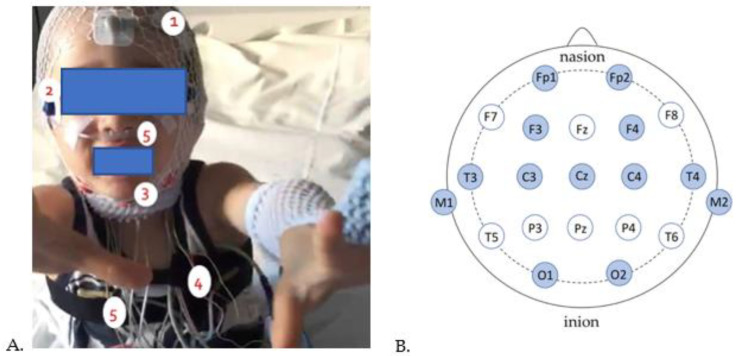
Pediatric subject fitted for polysomnography recording. (**A**) 1: Electroencephalogram (EEG), 2: Electrooculogram (EOG), 3: Electromyogram (EMG), 4: Electrocardiogram (ECG), 5: nasal pressure cannula and chest straps. (**B**) Schematic representation of EEG electrode placement on the scalp (in dark blue are the electrodes intended for our protocol). From front to back, the electrode letter labeling is as follows: Fp (pre-frontal or frontal pole), F (frontal), C (central line of the brain), T (temporal), P (parietal), and O (occipital).

The aim of polysomnography, performed here in a chronobiology room, is to study the stages of sleep and alertness (see Figure 1). All methods here follow the recommendations of the American Academy of Sleep Medicine [45].

In this study, we analyzed the following PSG measures: total sleep time (TST), sleep onset latency (SOL), wake after sleep onset (WASO), sleep efficiency, number of sleep stage changes (NSSC), as well as the durations, latencies, and percentages of the different sleep stages. The different sleep stages include light slow wave sleep (N1 and N2), deep slow wave sleep (N3), and REM sleep. Sleep fragmentation was measured mainly by the total arousal and micro-arousal index, WASO, sleep efficiency, and NSSC. The total micro-arousal index (index/TST) (index/h) is defined as a micro-arousal duration of more than 3 s and less than 10 s [45]. The total arousal index (index/TST) (index/h) is defined as the number of periods of wakefulness lasting more than one minute after the start of sleep.

### 2.3. Statistical Analysis

Statistical analyses were performed using Jamovi software (Version 0.9.5.12). The small number of subjects (n = 11) and controls and the absence of a normal distribution justified non-parametric analyses. We first described the data collected according to demographic and PTSD characteristics, followed by data on the sleep and circadian rhythms: qualitative data in terms of numbers and percentages and quantitative data in terms of mean and distribution. Spearman’s correlation analyses (non-parametric data) were then carried out within the PTSD subject group to explore the association between clinical PTSD parameters and objective PSG and actigraphy-derived sleep and circadian measures. Finally, we conducted non-parametric paired analyses (Wilcoxon signed-rank test) to compare sleep and circadian rhythms between PTSD and control subjects. Controls and PTSD subjects were matched on age and gender. A significance level of 5% corresponding to *p* < 0.05 (*: *p* < 0.05) was used.

## 3. Results

### 3.1. Characteristics of Subjective Parameters of PTSD, Sleep, and Circadian Rhythms in Children with PTSD

A total of 11 children with PTSD without intellectual disability were included in this study, including 8 girls and 3 boys, aged 3 to 17 years (mean age 12 years, SD 5 years) (see Table 1). Descriptive data on PTSD parameters revealed a mean score of 58.9 on the CPTS-RI scale, corresponding to severe PTSD. Subtype analysis showed that 60% of children had severe PTSD (CPTS-RI: 40–59), and 40% had very severe PTSD (CPTS-RI > 60). The CPC diagnostic scale confirmed these results, indicating that 100% of children have major functional repercussions, which is consistent with the CPTS-RI results. Analysis of the data relating to traumatic exposure (CPC) shows a single traumatic exposure for 27% of subjects, while 73% of subjects present repeated traumatic exposures. The nature of the traumatic event is variable, with 45% of traumatic exposure linked to war or terrorist attacks, 45% to sexual violence (rape), 27% to physical violence (abuse, acts of torture, and barbarism), and 54% of children with PTSD having witnessed traumatic events first-hand (for example, seeing a parent raped, killed or kidnapped). Finally, for 54% of children, the traumatic event involved several concurrent or systemic traumatic exposures.

The global subjective sleep assessment of PTSD children (SDSC) (see Table 1) indicates that all participants present sleep disturbances mainly within the insomnia and parasomnia spectrum, particularly nightmares (85.7% of children score positively on the SDSC for insomnia, 57.1% for parasomnia and 71.5% for daytime sleepiness). The NDQ-13 results (see Table 1) indicate the presence of nightmares associated with poor sleep quality for the entire population (100% of subjects). The characterization of nightmares gathered from semi-standardized questions shows a clear increase in the frequency of nightmares since the traumatic event for 44% of children, with several nightmare episodes per night for 77% of children. The content of the nightmares was directly related to the traumatic event (DSM-5 ‘intrusion symptoms’ criteria) in 100% of children. Nightmares are described as terrorizing (88% of children) and arousing (77%), with 88% of children remembering the nightmare on waking and 77% continuing to think about the nightmare ‘long after waking’. Finally, 100% of children described anxious hypervigilance at bedtime, with bedtime avoidance for 60% of children and bedtime requiring the presence of a family member for 40% of participants. For 88% of children, nightmares were accompanied by behavioral manifestations.

Not all parents could be evaluated, as some had died and/or the children were in care. Screening for psycho-traumatic reactions of the parents (n = 5) showed that all suffered from probable PTSD (TSQ score 7.2) (TSQ score 7.2). These parents all had poor sleep quality marked by moderate (25% of parents) or sub-clinical (75% of parents) insomnia and moderate anxiety (mean STAI score 53.2).

### 3.2. Characteristics of Objective PSG and Actigraphy Derived Sleep and Circadian Rhythm Parameters for Children with PTSD and Controls

Laboratory video-PSG results show no difference in sleep architecture (total sleep time, sleep latency, sleep efficiency) between PTSD children and controls (see Table 2), while the total microarousal index (14.8 for PTSD versus 8.2, *p* = 0.039) and the index of total microarousals and arousals (18.1 for PTSD versus 11.5, *p* = 0.042) are significantly increased for children with PTSD compared to controls, indicating a fragmentation of sleep.

**Figure 2:**
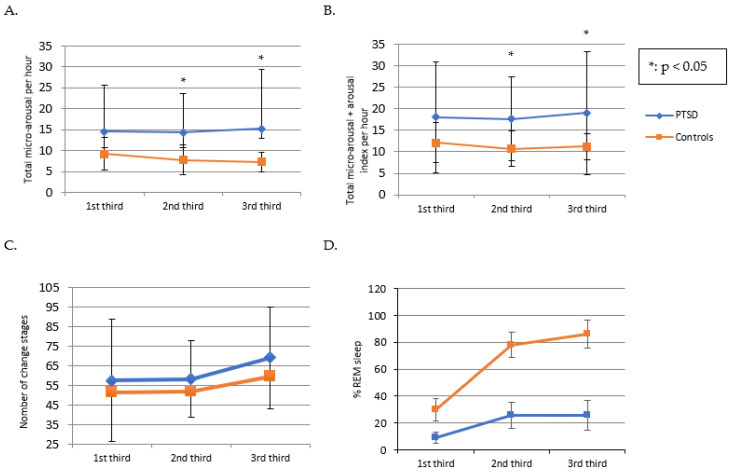
Comparison of sleep parameters by thirds of the night between PTSD children and controls. (**A**): Index of total micro-arousals per hour for each third of the night for PTSD subjects and controls. (**B**): Index of total microarousals and arousals per third of the night for PTSD subjects and controls. (**C**): Number of sleep stage changes per third of the night for PTSD subjects and controls. (**D**): Percentage of REM sleep per third of the night for PTSD subjects and controls. * *p* < 0.05. Error bars correspond to standard deviations.

Detailed analysis (by a third of the night) of sleep fragmentation parameters (micro-arousal index, total index of micro-arousals and arousals, number of sleep stage changes, and % REM sleep) provides information on the difference in mean values of these parameters between PTSD and controls for each third of the night. These analyses show that the difference in mean values between PTSD and controls increases significantly for the 2nd and 3rd thirds of the night (1st third of the night: 14.6 for PTSD versus 9.2 for controls (*p* = 0. 098), 2nd third of the night: 14.3 for PTSD versus 7.8 for controls (*p* = 0.039) and 3rd third of the night: 15.2 for PTSD versus 7.3 for controls (*p* = 0.020)) (see Figure 2A,B). Similarly, the difference between the mean values of PTSD and controls for the total index of microarousal and arousal increased towards the end of the night (1st third of the night: 18 for PTSD vs. 12.1 for controls (*p* = 0.652), 2nd third of the night: 17.6 for PTSD vs. 10.7 for controls (*p* = 0.020) and 3rd third of the night: 19 for PTSD versus 11.2 for controls (*p* = 0.004)). These results show a greater difference in sleep fragmentation (increase in micro-arousal index) in psychotraumatized subjects at the end of the night (see Figure 2A,B), with no significant difference in the number of sleep stage changes (184.1 for PTSD versus 158.5 (*p* = 0.11)) for PTSD and controls (see Figure 2C). In contrast, the percentage of REM sleep increases mainly for controls but not significantly (see Figure 2D). These trends suggest that sleep fragmentation in PTSD is more closely related to stage changes.

### 3.3. Correlation Analyses

Correlation analyses between subjective trauma and sleep data revealed an association between PTSD and nightmares (CPC diagnosis and NDQ-13 ‘fear’ component (r = 0.955, *p* < 0.001), total CPC and NDQ-13 ‘fear’ component (r = 0.882, *p* < 0.01), and CPTS-RI and NDQ-13 ‘fear’ component (r = 0.73, *p* < 0.05)), indicating a strong association between PTSD severity and the presence of post-traumatic nightmares. Furthermore, the association between the ‘fear’ component of the NDQ-13 and the ISI (r = 0.810, *p* < 0.05) indicates that children with PTSD and nightmares suffer more from insomnia symptoms. Moreover, for these children, the presence of nightmares has an impact on recovery, as shown by the correlations between the ‘interference’ component of the NDQ-13 (r = +1, *p* < 0.001) and the ‘premonition’ component of the NDQ-13 (r = 0.975, *p* < 0.01) with the ‘non-restorative sleep’ item of the SDSC. Finally, the association between CDI scores and the total SDSC score (r = 0.947, *p* < 0.05) shows that depressed children with PTSD have even poorer sleep quality. Overall, the subjective data show that for the population of children under study, there is a strong association between severe post-traumatic symptomatology, nightmares, and insomnia symptomatology, particularly for the component linked to neurovegetative activation (positive correlation with the ‘fear’ component of the NDQ-13).

Correlation analyses between subjective trauma, subjective sleep data, and objective PSG measurements show a strong association between insomnia symptoms (measured on the ISI) and PSG-derived TST in children with PTSD. Furthermore, the NDQ-13 ‘premonition’ (flashbacks) correlated with WASO (r = 0.946, *p* < 0.001), NSSC (r = 0.740, *p* < 0.05) and microarousal index (r = 0.732, *p* < 0.05), suggesting that the presence of post-traumatic nightmares is associated with sleep fragmentation parameters, underlining the link between post-traumatic nightmares and sleep fragmentation. Furthermore, there was an association between sleep efficiency and scores on the SDSC ‘non-restorative sleep’ (r = −0.074, *p* < 0.01), the SDSC total (r = −0.975, *p* < 0.01), and the CDI (r = −0.941, *p* < 0.01), indicating an association between lower sleep efficiency and the severity of sleep disturbances in PTSD subjects, and depression in the same subjects. Finally, there was a correlation between the parents’ STAI and the micro-arousal index (r = 0.943, *p* < 0.05) and the total arousal index (r = 0.886, *p* < 0.05), indicating either a link that could reflect the impact of parental anxiety on the child’s sleep, or the impact of the child’s sleep disturbances on the sleep of parents who were also possibly psychotraumatized, or an interplay of the two.

Overall, the results show an association between sleep fragmentation parameters and post-traumatic nightmares but no association under laboratory conditions between TST, sleep onset latency, sleep efficiency, and psychotrauma parameters. The laboratory results contrast the strong association between subjective measures of PTSD symptomatology, nightmares, and insomnia symptoms.

### 3.4. Sleep Quality under Controlled Compared to Ecological Conditions in Children with PTSD

TST, SOL, and sleep efficiency recorded at PSG in the laboratory were not different from controls but differed from objective sleep parameters in ecological at-home conditions (see Table 3). Indeed, psychotraumatized children had a shorter TST at home (actigraphy derived 479.6 min TST) compared to PSG in the laboratory (551.2 min PSG, *p* = 0.19), a higher sleep latency at home (32.4 min derived from actigraphy at home versus 18.9 min on PSG, *p* = 0.432) and significantly lower sleep efficiency at home (80.7% derived from actigraphy at home versus 90.8% on PSG, *p* = 0.014), all indicating that these children had significantly poorer sleep quality at home than in the controlled condition at the hospital.

**Figure 3:**
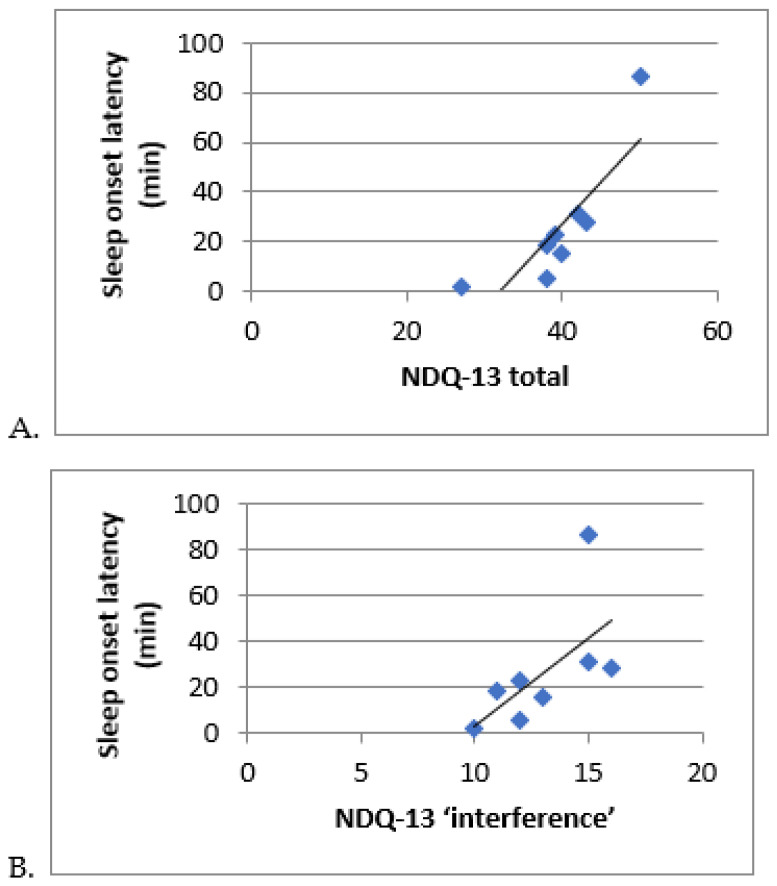
Correlation between actigraphy-derived sleep and rhythm parameters nightmares (NDQ-13). (**A**): Correlation between sleep onset latency and NDQ-13 ‘interference’. (**B**): Correlation between sleep onset latency and ‘total’ NDQ-13. NDQ-13: Nightmares distress questionnaire, version 13.

This trend is strengthened when comparing data derived from the same technique, actigraphy, performed during the night of hospitalization (laboratory conditions) with actimetry performed over 15 days at the children’s home since TST is significantly lower at home than when measured in hospital (464 min at home versus 586 min in laboratory, *p* = 0.018) and sleep latency is higher (79.21 min at home versus 25.64 min in laboratory, *p* = 0.55). Sleep latency measured by actimetry at home was associated with nightmares (NDQ-13 total: r = +0.886, *p* < 0.01 (see Figure 3A) and their functional repercussions (NDQ-13 interference: r = +0.771, *p* < 0.01 (see Figure 3B)). In Figure 3A,B, the extreme value at the top right corresponds to a young person with severely disturbed sleep, presenting a phase shift and a major sleep debt. In addition, sleep efficiency at home was associated with increased daytime sleepiness (SDSC sleepiness: r = +0.949, *p* < 0.05).

### 3.5. Characteristics of Circadian Parameters in Children with PTSD

Non-parametric circadian rhythm data (NPCRA) obtained from actigraphy carried out in the children’s homes show a mean L5 onset time (corresponding to the start of the five hours within 24 h when motor activity is minimal) at 03:12, indicating a possible phase shift (see Table 4). Furthermore, the inter-day stability (IS) is 0.48 (for normal values between 0 and 1), indicating variable rhythms from one day to the next.

L5 onset correlated with total CPC (r = −0.815, *p* < 0.05), suggesting a link between PTSD and phase shift. Furthermore, day-to-day rhythm variation (IS) is negatively associated with NDQ-13 (r = −0.756, *p* < 0.05) and insomnia (ISI: r = −0.755, *p* < 0.05). Associations between circadian parameters such as L5 onset, ISI and trauma parameters (CPC), and nightmares (NDQ-13 fear) suggest a link between circadian rhythm disturbances, PTSD, and nightmares.

## 4. Discussion

Our study shows frequent, severe, and disabling sleep disturbances, mainly within the spectrum of insomnia and nightmares in children with severe PTSD, with a strong association between the subjective complaint of sleep disturbances and posttraumatic symptomatology. These children with PTSD show (1) better sleep macro architecture under laboratory conditions (TST, SOL, and sleep efficiency) than under ecological conditions, (2) more fragmented sleep with altered sleep microarchitecture as assessed by in-hospital measurements (microarousal index, WASO, and number of sleep stage changes), as well as (3) disturbances of circadian rhythms. Sleep fragmentation parameters (4) are correlated with PTSD symptomatology, insomnia, and post-traumatic nightmares, especially for the component related to neurovegetative activation.

The children included in this study had particularly severe PTSD (see Table 1), which can be explained by the nature of the traumatic exposures and the fact that this was a clinical population recruited from regional psycho-trauma centers and university child psychiatry departments [3,46]. Indeed, the majority of children had been exposed to recurrent traumatic events concomitantly and in the context of double parent-child exposure, with parents also presenting posttraumatic symptoms. For these children, the higher rate of depression than in other studies may be explained by the severity and chronicity of PTSD in our population, as well as by associated sleep disturbances [17,47].

The frequency and severity of sleep disturbances (see Table 1) in our population of psychotraumatized children are consistent with existing literature data, particularly for insomnia [19]), nightmares, and poor sleep quality [6]. The prevalence of nightmares in our population is consistent with data from the literature, which describes between 50% and 80% nightmares one year after exposure to a traumatic event and up to 100% nightmares in the case of a major traumatic event, as is the case for our multi-exposed population [11,12]. Furthermore, the nightmare characteristics of our population are consistent with the results of other studies. Indeed, data from the literature show that the dreams of children who have experienced a traumatic event differ from those of other children in their content, notably in the high levels of negative emotions associated with them, the terrorizing atmosphere which frequently but not exclusively corresponds to replications of the traumatic event [10]. These nightmares, appearing in REM sleep but also in slow wave sleep, lead more systematically to nocturnal awakenings that can be associated with neuro-vegetative manifestations linked to ANS activation and maintenance of the memory on awakening [10]. As in other study populations, the nightmares of the children in this study are also associated with other sleep disturbances, in particular within the insomnia spectrum or other parasomnias (57.1% at SDSC parasomnia) [9,10]. In this regard, it should be noted that in our study, only 57.1% of children rated parasomnias on the SDSC, which is linked to the construction of the items on this scale, which focuses more on other parasomnias than on nightmares. These differences in scoring highlight the need to develop a measurement tool specifying post-traumatic nightmares as a complement to other parasomnias.

The association between insomnia and the ‘fear’ component (characteristic of ANS activation) of nightmares rated on the NDQ-13 (r = 0.810, *p* < 0.05) suggests that difficulties falling asleep are either related to neurovegetative symptoms (such as hyperarousal) linked to ANS activation [19], or secondary to nightmare-related sleep avoidance behaviors, or both. Furthermore, the association between the NDQ-13 ‘fear’ component and trauma symptoms at CPC diagnosis and CPTS-RI, and between NDQ-13 ‘fear’ and parasomnias at SDSC, insomnia at ISI, and non-restorative sleep provide further support for the hypothesis of ANS activation underlying both nightmares and PTSD, as well as their entanglement (see Results in Section 3.3). The existence of an association between post-traumatic symptomatology, sleep disturbances, and ANS activation is consistent with published data for other populations [48,49].

Parents’ sleep disturbances are consistent with changes in their child’s sleep since parents’ overall sleep quality is more deteriorated than sleep onset (subclinical insomnia on the ISI). This suggests that sleep deterioration is mainly related to their child’s nocturnal awakenings, as illustrated by the correlations between the parents’ STAI and the child’s microarousal index and arousal index (see Results in Section 3.3). The parent’s level of moderate anxiety is probably more related to concern for the child’s condition than to the parents’ post-traumatic symptomatology, with either an impact of the parents’ anxiety on the child’s sleep or an impact of the child’s sleep disturbances on the sleep of the equally psycho-traumatized parents, or an interplay of the two [50].

The lack of difference in objective sleep macroarchitecture parameters (PSG-derived TST, sleep efficiency, and sleep latency) between children with PTSD and controls (see Table 2) contrasts with the expected results found in the literature (decreased TST and sleep efficiency and increased sleep onset latency [19]), and with evidence from the children’s subjective assessments (see Table 1), which highlight (i) insomnia on falling asleep, (ii) reduced sleep time (44% of the population rated TST between 5 and 7 h on the SDSC), (iii) a strong correlation between PTSD symptomatology and nightmares and insomnia, and (iv) the existence of a correlation between TST measured on the PSG and subjective assessment of insomnia on the ISI. Nevertheless, despite better sleep during inpatient recording, sleep fragmentation with changes in sleep microarchitecture (significant difference in the micro-arousal index and total micro-arousal and arousal index between PTSD and controls in the second half of the night and at the end of the night, increase in WASO and increase in number of stage changes) was still evidenced under these conditions. Moreover, data collected under ecological conditions showed significant sleep alterations, with a significantly shorter at-home actigraphy-derived TST compared with inpatient actigraphy and a significantly lower sleep efficiency on home actigraphy compared with inpatient PSG (see Table 3).

These data reveal an exceptionally high quality of sleep perceived and reported in hospital conditions compared with ecological conditions. They may reflect the impact of a secure environment on sleep and post-traumatic symptomatology, as illustrated by the association between insomnia, falling asleep at home, and nightmares (see Figure 3A,B). In addition, circadian data (derived from home actigraphy) showed low inter-day stability (IS at 0.48), indicating a relatively unstable sleep-wake rhythm reflecting alternating short nights and nights of recovery. The association of IS with nightmares and insomnia (see Results Section 3.5) suggests that the disruption of sleep-wake rhythms is related to traumatic reactivations linked to external environmental factors. These results demonstrate the importance of experimental conditions (evaluation context and temporality) for these highly environmentally-reactive populations. PSG is the most accurate tool for measuring sleep. However, these measurements have their limitations, as they are usually carried out over short periods and in a controlled environment, whereas actigraphy can be conducted under ecological conditions, though it does not allow us to explore sleep in itself but rest-activity rhythms. Actigraphy is more representative of the subject’s actual sleep-wake rhythms, whereas PSG enables precise exploration of sleep architecture. In this context, the night of hospitalization can be seen as a ‘night of recovery’, favored by the impact of the experimental conditions (controlled environment, containing chronobiological recording chambers, privileged relational time with the parent and nurses, presence of a competent and reassuring health professional reinforcing the feeling of security, video-camera, recognition of the child’s difficulties via an objective assessment, etc.) and their reassuring effect on this population. In this respect, the specificity of chronobiological recording chambers, designed specifically to attenuate noise and be globally hypo-stimulating at the sensory level, enabled a reduction in stress levels for this particular hypervigilant population. This environment, possibly attenuating ANS activation, probably helped avoid the first-night effect described in the literature [19,51]. In fact, for some children, home is the place where the aggressions were committed against them or other family members living with them. What is more, our child psychiatric population is very different from the populations usually recorded, which are adults, mainly male, military and/or war veterans, with multiple associated comorbidities impacting sleep (metabolic, cardiovascular, substance use and/or sleep disturbances or psychiatric such as obstructive sleep apnea syndrome) [19,51]. Indeed, existing smaller studies on civilian adult populations (road traffic accidents) or for ‘early’ PTSD highlight a TST and SOL similar to control populations [18,21], as was found in our study (see Table 2).

The fragmentation of sleep (see Figure 2A,B) in psychotraumatized children is consistent with other work that also highlights an increase in the microarousal and arousal index [19,20] and an increase in WASO [19,20]. In our study, the increase in sleep micro-fragmentation at the end of the night (see Figure 2A,B) is probably more linked to changes in sleep stages (see Figure 2C) than to REM sleep (see Figure 2D) [18,52]. Moreover, the modification of sleep microarchitecture would potentially be linked to ANS activation, as suggested by the association between sleep fragmentation and posttraumatic nightmares: association between the microarousal index and NDQ-13 ‘premonition’, between NCS and NDQ-13 ‘reviviscences’ and between WASO and NDQ-13 ‘reviviscences’ and WASO (see Results Section 3.3). Furthermore, the highly significant correlations between the NDQ-13 ‘fear’ component and the trauma parameters at CPC and CPTS-RI provide further support for the hypothesis of ANS activation underlying both nightmares and PTSD and their entanglement.

In addition, we know that the pathophysiology of post-traumatic nightmares is linked to the state of heightened wakefulness during the day and night and the impossibility of extinguishing the learned fear [53]. Thus, post-traumatic nightmares can lead to difficulty in falling asleep and maintaining sleep with an awareness of nocturnal awakenings that can reinforce erroneous beliefs about sleep as responsible for insomnia [53]. Moreover, we know that post-traumatic nightmares occur in both REM and slow wave sleep [10] and that post-traumatic nightmares characterized by an exact reproduction of the traumatic scene are more correlated with the onset of PTSD [54], which has led some authors to postulate that recurrent nocturnal vision of the traumatic scene would constitute repeated re-exposures that would consolidate the traumatic memory. Finally, the alternation of slow-wave and REM sleep during the night plays a major role in memory consolidation and the processing of emotional information. In this respect, various studies have shown an association between micro-arousals and arousals on the one hand and anxiety and panic symptoms on the other, suggesting a link with ANS hyper-activation and emotional hypo-modulation [19,20]. Thus, the sleep fragmentation highlighted in our study could participate in the chronicization of PTSD due to the absence of emotional extinction, which would explain the role of sleep disturbances in post-traumatic symptoms and their increase. Moreover, it is known that emotional hyporegulation (increased anxiety levels) can be reinforced by sleep debt.

In our population, the majority of psychotraumatized children do not have their recommended sleep duration for their respective age (81.9% sleep less than the recommended duration, based on home actigraphy recording) (National Sleep Foundation, 2015) [55]. Sleep debt (1 h38 as per CCTQ) in children is associated with trauma (CPTS-RI: r = 0.847, *p* < 0.05) and daytime sleepiness in our subjects (sleep efficiency and SDSC sleepiness: r = 0.949, *p* < 0.05; sleep efficiency and SDSC ‘non -restorative sleep’ (r = −0.074, *p* < 0.01). However, current scientific data show that sleep deprivation induces a reduction in connectivity between the prefrontal cortex and the amygdala, which increases amygdala hyperactivity [56] and, ultimately, ANS activation. Clinically, this translates into deficits in emotional and physiological regulation and an increase in posttraumatic symptoms and sleep disturbances [57]. In addition, other studies show that sleep fragmentation and increased sympathetic activity during sleep are predictive of the development of PTSD [21].

Non-parametric assessment of circadian rhythms shows a phase shift (L5 onset at 3:12 a.m.) (see Table 4) at home, which correlates with posttraumatic symptoms (see Results Section 3.5). One hypothesis is that this phase shift would be of psychological and behavioral origin due to powerful post-traumatic avoidance processes [3]. These links between post-traumatic symptoms and circadian parameters warrant further exploration, particularly in adolescent populations who are undergoing a physiological phase shift [41,58] or in subjects with evening chronotypes. Indeed, although the effect of chronotype as a moderator of associations between sleep parameters and post-traumatic stress was not significant, ‘being of the evening’ was associated with higher post-traumatic scores in psychotraumatized adult populations, confirming the vulnerability of ‘evening people’ to more severe stress reactions [25].

The main limitation of our study is its small sample size and the wide age range of the pediatric subjects (3 to 18 years). In addition, at this stage, we were unable to include a control population for the at-home actigraphy. Furthermore, the lack of variation in REM sleep distribution could have been complemented by other measures, such as the mean duration of continuous REM sleep, which could be more correlated with the severity of PTSD or the number of REM sleep periods [21], or even the density of eye movements during REM sleep [18,22,51]. Finally, the interpretation of our results is complicated by the fact that some variables are correlated with each other. Future teams working on this subject will need to use larger populations with cross-validation to avoid over-fitting.

On the other hand, the significant strengths of the study are that it is one of the few studies to subjectively and objectively (using PSG and actigraphy) assess sleep and circadian rhythms in children with PTSD, both under laboratory and ecological conditions. This dual exploration is important because psychotraumatized populations are highly reactive to their environment. Furthermore, the children included in the study correspond to a clinical population that has been exposed to different types of trauma, which is representative of the patients currently cared for in hospital structures, increasing the validity of our study results for patient populations.

## 5. Conclusions

This pilot study revealed severe and disabling sleep disturbances, mainly within the spectrum of insomnia and nightmares, in children with severe PTSD, compared to controls, with a strong association between the subjective complaint of sleep disturbances and posttraumatic symptomatology. Results were obtained assessing sleep and circadian rhythms subjectively and objectively under controlled and ecological conditions. Children with PTSD displayed a better sleep architecture in controlled conditions (hospitalization and sleep laboratory) than in ecological conditions, but a more fragmented sleep (increased WASO, increased number of sleep stages) with a modified sleep microarchitecture (increased micro-arousal index), as well as associations between sleep fragmentation parameters and posttraumatic nightmares on the one hand, and PTSD symptomatology and nightmares and insomnia on the other. Our results reinforce the hypothesis of a bidirectional link between sleep disturbances, particularly posttraumatic nightmares, and PTSD symptoms, which may be underpinned by common pathophysiological processes such as ANS dysregulation. Circadian data suggest that psychotraumatized children may present circadian rhythm disturbances in the form of phase shifts.

Our study shows the importance of a systematic assessment and treatment of sleep and rhythm disturbances in children with PTSD and underlines the importance of assessments under ecological conditions. The use of portable devices for measuring sleep-wake rhythms combined with measurements of autonomic nervous system activation parameters (heart rate variability) should be used in order to investigate the participation of ANS dysregulation. Portable devices would allow both (1) to obtain data under ecological conditions measuring the impact of environmental conditions on the sleep of children with PTSD and on PTSD symptoms, in particular by exploring the impact of external traumatic reactivation factors (sleep environment, proximity to the trauma site, reactivation linked to specific events such as the approach of a trial, reactivation linked to family cohabitation in the event of joint traumatic exposure, etc.), but also (2) to study the physiopathology and the links between dysregulation of the ANS, posttraumatic symptoms and sleep disturbances (insomnia and nightmares) with the aim of developing appropriate therapeutic strategies.

In the future, bifocal therapies targeting both sleep and trauma and integrating analyses of the living environment (sleep environment design, sleep hygiene, control of environmental elements of traumatic reactivation, etc.) into care should be developed and offered to psychotraumatized children and their parents, in addition to specific care for sleep disturbances and trauma. In this respect, the development and evaluation of targeted care for children with sleep disturbances associated with PTSD is necessary, whether through psychotherapeutic approaches (mental imagery rehearsal therapy targeting post-traumatic nightmares; exposure, relaxation, and rescripting therapy) or pharmacological approaches (prazosin for post-traumatic nightmares, melatonin, etc.). All of these treatments, combined with adjustments to the sleep environment, may help children with PTSD and their parents achieve more restful nights, avoiding the common sleep debt that contributes to maintaining post-traumatic symptoms and sleep disturbances. The development of systemic therapeutic approaches, including care for parents, should also be encouraged. Finally, secondary prevention interventions to help maintain quality sleep directly after traumatic exposure should also be an issue in the future.

## Figures and Tables

**Table 1:** Subjective parameters of PTSD, sleep and circadian rhythms for children with PTSD.

Description of PTSD Population (n = 11)							
Variables	Mean	Median	Standard Deviation	Minimum	Maximum	Scale Cut-Off	% Population with Score+
Child trauma assessment							
CPTS-RI total	58.9	58	5.9	51	67	_	100
Mild PTSD	_	_	_	_	_	12–24	0
Moderate PTSD	_	_	_	_	_	25–39	0
Severe PTSD	58.9	58	5	43	58	40–59	60
Very severe PTSD	62	63	2.5	61	67	>60	40
CPC total	62.9	64	18.1	32	93		
CPC PTSD diagnosis	52	48.5	18.2	22	83	>20	100
CPC functional repercussions	12.8	13	5.3	4	20	>4	100
Child sleep assessment							
ISI total	18.3	18	4	12	25	Moderate insomnia > 15	90
NDQ-13 total	37.9	39	7.9	24	50	>20	100
NDQ-13 fear items	17.3	19	4.8	8	24	>10	90
NDQ-13 interference items	12.6	12	2.4	9	16	>7	100
NDQ-13 premonition items	4.8	4	1.6	3	7	>4	80
SDSC total	61.1	62	7.2	47	68	>45	100
SDSC insomnia	23.2	24.5	4	15	27	>21	85.7
SDSC parasomnia	17.3	17	3.8	13	23	>17	57.1
SDSC respiratory disturbances	8.8	8.5	1.7	7	11	>12	0
SDSC non-restorative sleep	6.9	7	2.1	3	10	>11	0
SDSC daytime sleepiness	5	5	0.8	4	6	>5	71.5
Children’s comorbidities							
Children’s CDI	28.1	27.5	4.8	23	35	_	100
Description of parent population (n = 5)							
Evaluation trauma parents							
TSQ total	7.2	8	2.1	5	10	>5	100
Parents sleep assessment							
ISI	14.7	13.5	5.12	10	22	Moderate insomia > 15	25
PSQI	12.7	13	0.6	12	13	>5	100
STAI parents	53.2	59	11.7	33	66	Moderate anxiety > 46	87.5

Data are presented as scores for each scale, and quantitative variables as mean and median (+/− standard deviation). Numbers with a positive score in % of the study population. PTSD: Post-Traumatic Stress Disorder; CPTS-RI: Child Post-Traumatic Stress Reaction Index; CPC: Child PTSD Checklist; ISI: Insomnia Severity Index; NDQ-13: Nightmare Distress Questionnaire Version 13; SDSC: Sleep Disturbance Scale for Children; CDI: Child Depression Index; TSQ: Trauma Screening Questionnaire; PSQI: Pittsburgh Sleep Questionnaire Index; STAI: State-Trait Anxiety Inventory.

**Table 2:** Description of PSG characteristics of pediatric PTSD population and controls.

Description of PSG Characteristics of PTSD Population and Controls			
Variables	PTSD (n = 11)	Controls (n = 11)	*p*
Total Sleep Time, TST (min)	565.6 (±117.7)	557.3 (±64.5)	0.82
Sleep Efficiency (TTS/PTS × 100) (%)	90.1 (±6.4)	93.6 (±4.3)	0.30
Sleep efficiency (%)	86.6 (±7.8)	88.0 (±5.8)	0.82
Sleep latency, LE (min)	21.7 (±19.3)	22.1 (±23.6)	0.91
Time awake after sleep, WASO (min)	62.2 (±35.6)	52.3 (±29.6)	0.65
Total sleep time, DTV (LE + WASO) (min)	59.2 (±35.6)	38.2 (±27.9)	0.30
Number Changes Stages	184.1 (±70.7)	158.5 (±87.4)	0.11
% N1	8.6 (±6.7)	3.9 (±2.4)	0.08
% N2	43.6 (±10.6)	43.5 (±13.0)	0.73
% N3	25.0 (±11.1)	26.4 (±10.5)	0.86
REM	20.9 (±5.0)	24.3 (±6.6)	0.77
% Total NREM	78.1 (±5.0)	74.7 (±6.6)	0.77
Total micro-arousals index (index/h)	14.8 (±10.8)	8.2 (±2.5)	0.039 *
Total arousals index (index/h)	13.3 (±1.5)	12.6 (±13.9)	0.06
Index micro-arousals + arousals total (index/h)	18.1 (±11.5)	11.5 (±3.2)	0.042 *
Average HR TTS (bpm)	71.6 (±10.6)	76.1 (±11.9)	0.21

Data presented as means (±standard deviation) for the different values. PTSD: posttraumatic stress disorder; WASO: wake after sleep onset; TTS: total sleep time N1%: percentage of light slow wave sleep at sleep onset, stage 1 N2%: percentage of light slow wave sleep, stage 2 N3%: percentage of deep slow wave sleep, stage 3 SP%: percentage of REM sleep; PM: periodic movement; REM: rapid eye movement; NREM: non-rapid eye movement; HR: heart rate. *: *p* > 0.05.

**Table 3:** Comparison of PSG sleep data, laboratory, and home actimetry for children with PTSD.

Comparison of Sleep Parameters from PSG and Actimetry in Laboratory and Home Conditions for Children with PTSD					
	PSG in the Laboratory	Actimetry in Laboratory	Home Actimetry	Home/Laboratory Actimetry	PSG/Home Actimetry
Variables	Average	Average	Average	*p*	*p*
TST (min)	585.6	586.6	464.3	0.018 *	0.19
Efficacity (%)	90.8	86.6	79.2	0.17	0.014 *
SOL (min)	18.9	25.6	79.2	0.55	0.43

Data are presented as mean values. TST: total sleep time; SOL: sleep onset latency. * *p* > 0.005 (Paired analysis, Wilcoxon).

**Table 4:** Subjective and actigraphy-derived objective circadian rhythm data for children with PTSD.

Circadian Data for PTSD			
Variables	Mean	Standard Deviation	Median
Objective circadian data (NPCRA)			
L5 onset (h)	03:12:00	06:18:00	01:30:00
M10 onset (h)	09:30:00	01:18:00	09:30:00
RA (Relative Amplitude)	0.91	0.05	0.91
IS (Inter-daily stability)	0.48	0.13	0.45
IV (Intraday variability)	0.73	0.15	0.67
Subjective circadian data CCTQ)			
MSFsc	3.10	1.39	2.82
Total MSFsc (h)	03:05:49	01:23:17	02:35:54
Sleep debt (h)	01:38:00	00:47:00	02:16:00
Sleep debt (% of total)	50		

Values given as mean, standard deviation and median. NPCRA: Non-Parametric Circadian Rhythm Analysis; L5 onset: start of five-hour nycthemer with minimum activity; M10 onset: start of ten-hour nycthemer with maximum activity; IS: inter-day stability; IV: intra-day variability; RA: relative amplitude; CCTQ (Child Chronotype Questionnaire); MSFsc (Middle Sleep on Free days corrected) = MSF − 0.5 (TTS on free days-TTS on work days (MSF (Middle Sleep on Free days) = sleep onset time on free days + (TTS on free days/2)); Neutral chronotype: MSFsc: 2.17–7.25; Morning chronotype ≤ 2.17; Evening chronotype: MSFsc ≥ 7.25; Sleep debt: difference between TTS between free days and school days greater than 2 h.

## Data Availability

The data presented in this study are available on request from the corresponding author.
